# The A B C Medical Diary and Visiting List

**Published:** 1889-12

**Authors:** 


*The A B C Medical Diary and Visiting List.* 1890. London: BURROUGHS, WELLCOME, & CO.

To the convenience of an ordinary visiting-list, this adds very full information about recently-introduced drugs. This is not only most helpful to the prescriber, but is a testimony to the enterprise of the noted firm to whom the profession is indebted for many practical reforms in the administration of drugs. An elegant wallet, serving many useful purposes, is supplied as an extra cover for the book.

